# A Typical Case of an Atypical Disease: Klatskin Tumor

**DOI:** 10.7759/cureus.28782

**Published:** 2022-09-04

**Authors:** Vartika Agrawal, Yeshwant Lamture, Sangeeta Totade

**Affiliations:** 1 Medicine, Jawaharlal Nehru Medical College, Datta Meghe Institute of Medical Science, Wardha, IND; 2 Surgery, Jawaharlal Nehru Medical College, Datta Meghe Institute of Medical Science, Wardha, IND; 3 Pharmacology, Jawaharlal Nehru Medical College, Datta Meghe Institute of Medical Sciences, Wardha, IND

**Keywords:** surgical resection, ca-19-9, intrahepatic disease, biliary tract tumor, cholangiocarcinoma

## Abstract

Cholangiocarcinoma is a rare type of cancer disorder. The case report is of a 55-years-old male patient who came to the output patient department (OPD) with complaints of abdominal pain and weight loss for 15 days and later was diagnosed with Klatskin tumor after mandatory investigations. After the approval of the tumor board committee, he is under chemotherapy, to which he is responding positively. Klatskin tumor is a type of cholangiocarcinoma occurring at the convergence of the right and left lobes of hepatic bile ducts, forming the common bile duct. The source of cholangiocarcinoma is idiopathic, and most cholangiocarcinoma is treated with the help of either surgical resection or chemotherapy. Surgical resection is performed in initial cases, but most patients present with the advanced stage of Klatskin tumor. CA-19-9 is a tumor marker that indicates the presence of a Klatskin tumor.

## Introduction

Klatskin tumor is a cholangiocarcinoma (CCA) that occurs at the intersection of the right and left lobes of hepatic bile ducts forming the common bile duct. The disease is named after Gerald Klatskin, who in 1965 elaborated on 15 cases, and features were found similar to cholangiocarcinoma [1]. The source of cholangiocarcinoma is idiopathic. Numerous pathologic conditions, substantial in any severe or long-lasting biliary tract epithelial damage, can be a little hostile. Risk factors associated with cholangiocarcinoma are: Primary sclerosing cholangitis, which consists of 40% of the cholangiocarcinoma, is due to an unidentified provocative disorder of the biliary tree; 25% of cases are associated with genetic biliary cystic diseases, like choledochal cysts or Caroli's disease; these are more invasive than others. There is also an association of reflux of pancreatic secretions into the bile duct, which happens because of an asymmetrical pancreaticobiliary duct junction. Liver flukes such as Clonorchis sinensis and Opisthorchis viverrine have also been recognized as a prevalent cause of chronic disease-causing biliary tract infection in the Southeast Asian population. Moreover, manufacturing contact with asbestos and nitrosamines, and regular exposure to the radiologic agent, thorium dioxide, are also related to the progress of cholangiocarcinoma [2,3]. Cholangiocarcinoma can be divided into three categories based on scans: Perihilar or Klatskin tumors, Intrahepatic CCA, and Extrahepatic CCA.

The spread of tumors in bile ducts can be divided into the following types according to the classification of Bismuth-Corlette, which are as follows: Type I: tumors below the convergence of the left and right hepatic ducts; Type II: tumors reaching the junction but not including the left or right hepatic ducts; Type III: tumors obstructing the common hepatic duct and either the right or left hepatic duct; Type IV: multicentric tumors involving the convergence and the right and left hepatic ducts [4-7]. The second most malignant neoplastic change in liver tumors occurs in cholangiocarcinoma. It is mainly due to the invasive conversion and growth of the biliary duct epithelium. Cholangiocarcinoma is a rare adenocarcinoma with an underprivileged prognosis. Although the one-year survival has improved supplementarily, the five-year subsistence has not discovered any substantial modification (less than 5%) [6]. Cancer in the biliary tract junction is called hilar cholangiocarcinoma (HC) or Klatskin tumor, a malignant disease. It is a rare condition as it comprises 10-20 % of the total intrahepatic disorders, while it is among 2% of all cancers. The prognosis is abysmal, and most Klatskin tumors are believed to be unresectable upon diagnosis [8]. This case report is being put forward to offer information regarding Klatskin tumor, its clinical presentation, investigation, and diagnosis, followed by treatment of such rare cases. This case report will be helpful to the medical sciences for future instances of the same.

## Case presentation

A 55-year-old male came to the outdoor patient department (OPD) complaining of jaundice for 15 days, weight loss (approximately 2 kgs) over the past 15 days, and generalized weakness for 15 days. The patient was well oriented with time, place, and person. On general examination of the patient, his respiratory rate was 18 breaths/min, oxygen saturation (SpO2) level was 98%, Blood pressure 110/70 mmHg, Glasgow coma scale (GCS) 15/15, and random blood sugar was 153 mg/dl. The patient gave a history of dark-colored urine. Pallor was present in the patient while he had a negative history of icterus, cyanosis, clubbing, lymphadenopathy, and edema. The patient presented no history of abdominal pain, constipation, loose stools, fever, backache, and cough/breathlessness. The patient is not recognized with chronic disorders like diabetes mellitus, hypertension, tuberculosis, or bronchial asthma. Also, there was no other significant surgical history in the past. Also, the patient is not a known case of Hepatitis B and human immunodeficient virus. On palpation, there was the presence of hepatomegaly.

Radiologic investigation results

Magnetic Resonance Cholangiopancreatography (MRCP)

**Figure 1:**
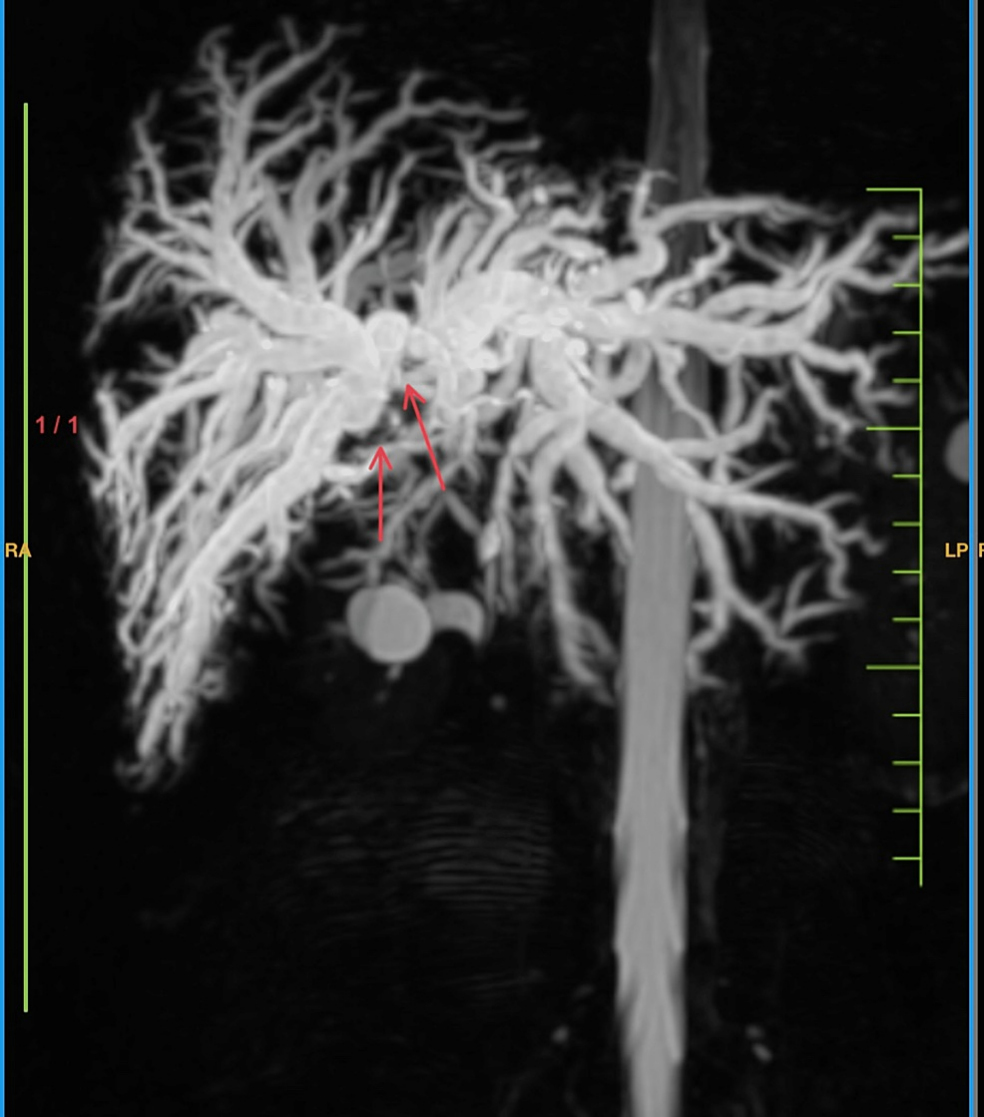
Magnetic resonance cholangiopancreatography Arrows represent the grossly dilated intrahepatic and biliary radicles also there is significant dilation of the right and left hepatic duct

MRCP reveals the biliary hilar butterfly-shaped infiltrating neoplastic mass lesion which causes the gross dilatation of right and left hepatic ducts resulting in dilatation of intra-hepatic biliary radicles which indicates Type IV Bismuth (Figure 1).

Ultrasonography (USG) Whole Abdomen

**Figure 2:**
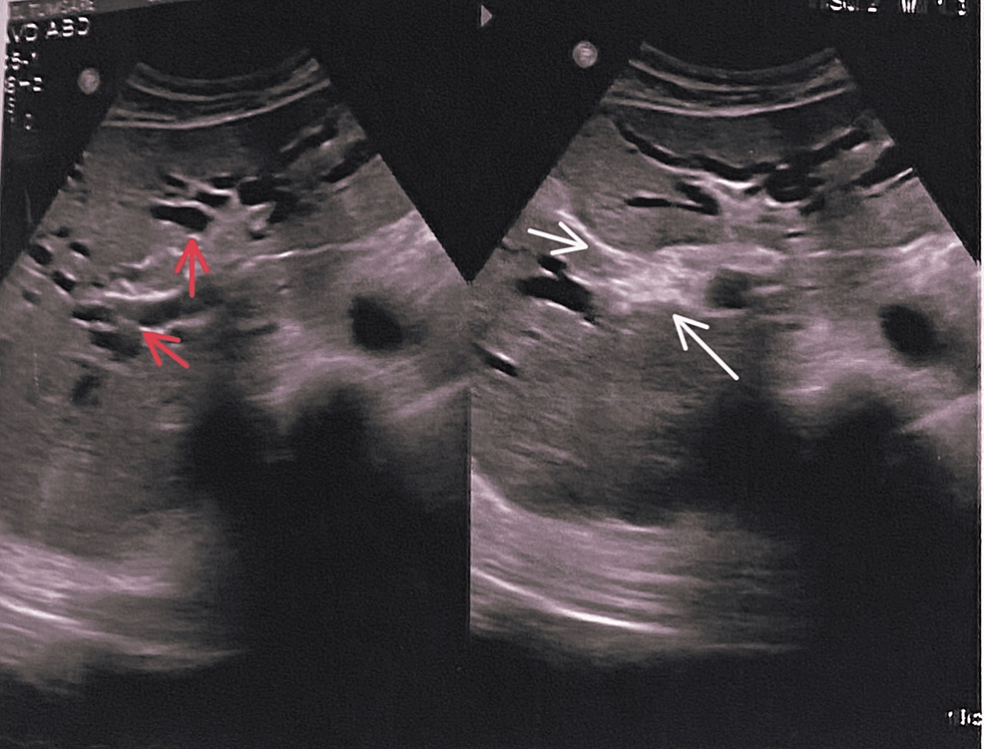
Ultrasonography of the whole abdomen Red arrows represent the dilated veins while the white arrow shows the obstructive dilatation of intrahepatic biliary radicals

Ultrasonography (USG) whole abdomen reveals mild hepatomegaly with the possibility of neoplastic lesion involving biliary hilum and congestive heart defect (CHD) causing obstructive dilatation of intrahepatic biliary radicals (IHBR). This finding led to a suspicion of cholangiocarcinoma/Type II lesion as shown in Figure 2.

Contrast-Enhanced Computed Tomography (CECT)

**Figure 3:**
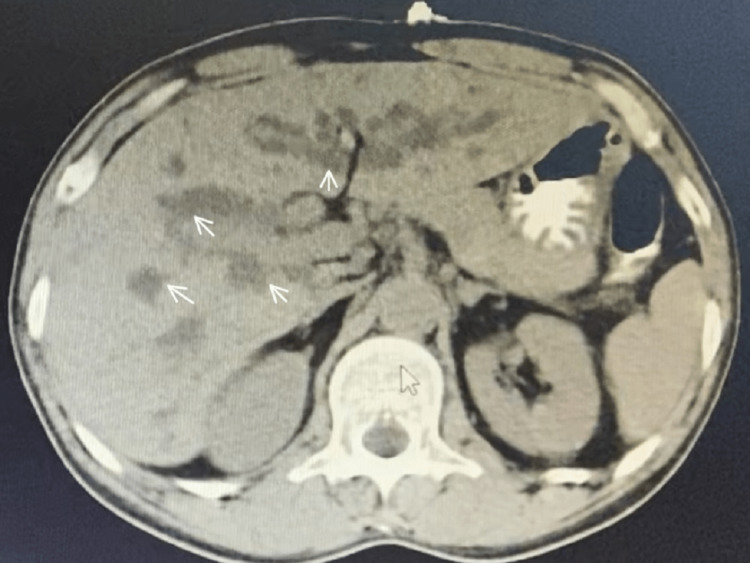
CECT scan of the abdomen CECT: Contrast-enhanced computed tomography, IHBR: Intrahepatic biliary radicals White arrows represent the grossly dilated IHBR

Grossly dilated IHBR, right and left hepatic ducts with no obvious hepatic or hilar mass lesion periductal infiltration or intraductal cholcarcinoma were observed as shown in Figure 3. 

Table 1 shows the laboratory diagnosis results of the patient's blood. The patient was transferred to the in-house cancer hospital as a suspected Klatskin tumor case supported by the findings of the above investigations. His case was later analyzed and recognized as a Klatskin tumor case. Now the patient is under chemotherapy and is responding positively to the treatment.

**Table 1:** Laboratory diagnosis results

Investigations	Observed value	Biological reference range	Unit
Total Bilirubin	1.2	0.2-1.2	mg/dl
Direct Bilirubin	13.19	0.0-0.30	mg/dl
Indirect Bilirubin	8.87	0.1-0.7	mg/dl
Serum glutamic-oxaloacetic transaminase (SGOT)	145	0-40	IU/L
serum glutamic-pyruvic transaminase (SGPT)	129	0-42	IU/L
Alkaline phosphatase	400	17-406	IU/L
Total Protein (TP)	6.9	6.6-8.7	gm/dl
Albumin	2.6	3.6-4.4	gm/dl
Globulin	3.13	0.0-3.5	gm/dl
Serum Creatinine	1.20	0.5-1.30	mg/dl
Tumor marker CA-19-9	0.2	0.0-37	U/ml
Hemoglobin (Hb)	11.6	13.2-16.6	gm/dl
Prothrombin Time (PTT)	3.5	11-13.5	sec

## Discussion

This case report is not only regarding the rare case of Klatskin tumor but also aims for the medical sciences to publish more articles on this topic. Until now, only a few cases regarding cholangiocarcinoma have been published. Some of the comparable aspects of cholangiocarcinoma reports with published cases are included here. In one of the reported cases, Giron and Alcantar reported that CA-19-9 levels become high in advanced cholangiocarcinoma [9]. But in the present case, the CA-19-9 level is not elevated. But, the patient is presenting with increased levels of liver enzymes and the presence of obstructive jaundice symptoms. Varda et al., in one of the cases, stated that the typical presentation of disease includes the presentation of symptoms of obstructive jaundice along with the loss of weight or onset of diabetes mellitus [10]. Also, the utmost precise indicator of IgG4 cholangiopathy is elevated in this disease. But in this case, there is no significant rise in serum IgG4, which is then demonstrated in infrequent patients. Ayas et al., in one of the cases, observed that cholangiocarcinoma is a rare tumor with a poor prognosis and complex treatment, making it an undiagnosed entity [11]. But, in this case, the issue was identified as soon as he presented with the disease in the hospital, which led to better chances of responding to the provided treatment. Also, the patient is undergoing chemotherapy as the diagnosis was made swiftly, and the patient responded positively to the treatment. One of the research projects by Barner-Rasmussen et al. stated that the prevalence of the disease is comparatively more in males than females [12]. It also depends upon the patient’s age; as the patient's age increase, the chances of disease causation increase [13]. Cardinale et al. stated in one of their case reports that tumors can be classified under two categories: Intra-hepatic CCA (IH-CCA) and Extra-hepatic CCA (EH-CCA). IH-CCA develops within the liver epithelium, while EH-CCA mainly occurs in the biliary tract [14]. Thus, according to the tumor’s position, in the present case tumor lies in the EH-CCA.

## Conclusions

Klatskin tumor is the presentation of a rare disease. Usually, the presentation of this disease is in an advanced stage, but the diagnosis and treatment of these tumors will have better consequences in the early stage of these tumors because surgical removal of the tumor can be performed in the initial cases. Also, a lot of investigation methods such as MRCP, USG, and CT scans can be helpful for the better diagnosis of a patient. The present report is a Klatskin tumor case. The patient in the reported case is undergoing chemotherapy after proper analyses, diagnosis, and approval by the tumor board committee. After starting the therapy, the patient is responding to the treatment. The publication of such cases will be helpful to the medical sciences for early detection and diagnosis of the disease, helping them provide people with better health care. Thus, improving the health ratio of the society. The primary cause of failure in early diagnosis of this disease is because of the presentation of these conditions in advanced stages, where the survival chances of patients are less.
